# Utility of hepatic or total body iron burden in the assessment of advanced hepatic fibrosis in HFE hemochromatosis

**DOI:** 10.1038/s41598-019-56732-0

**Published:** 2019-12-27

**Authors:** Justin Chin, Lawrie W. Powell, Louise E. Ramm, Oyekoya T. Ayonrinde, Grant A. Ramm, John K. Olynyk

**Affiliations:** 1 Department of Gastroenterology & Hepatology, Fiona Stanley Fremantle Hospital Group, Murdoch, Western Australia Australia; 2 Faculty of Medicine, The University of Queensland, Herston, Brisbane, Queensland Australia; 3 Hepatic Fibrosis Group, QIMR Berghofer Medical Research Institute, Herston, Queensland Australia; 4 School of Medical and Health Sciences, Edith Cowan University, Joondalup, Western Australia Australia

**Keywords:** Liver cirrhosis, Liver fibrosis

## Abstract

Development of advanced hepatic fibrosis in HFE Hemochromatosis (HH) is influenced by hepatic iron concentration (HIC) and age. In patients with HH, it is important to assess the likelihood of cirrhosis and thus the need for confirmatory liver biopsy. Therapeutic phlebotomy also provides an estimate of mobilisable iron stores. We determined whether mobilisable iron stores may predict the presence of advanced fibrosis. Retrospective analysis of 137 male and 65 female HH subjects was undertaken. Biochemical, histological and phlebotomy data were available on all subjects. The mean values of HIC, HIC × [age], mobilisable iron, mobilisable iron × [age] and serum ferritin in the cohort were higher in the group with advanced fibrosis. HIC had an optimum sensitivity and specificity of 73% for the diagnosis of advanced liver fibrosis, with a cut-off HIC level of 200 µmol/g (AUROC 0.83, p < 0.0001). AUROC for HIC was greater in females (0.93) than males (0.79). Mobilisable iron had an optimum sensitivity and specificity both of 83% at a cut-off of 9.6 g for the prediction of advanced fibrosis in all subjects (AUROC 0.92, p < 0.0001). Mobilisable iron stores provide a simple, clinically useful indication of the risk of advanced fibrosis and should routinely be considered.

## Introduction

HFE Hemochromatosis (HH) is an autosomal recessive disorder of iron metabolism and is one of the most common genetic disorders in Caucasian populations, with a prevalence of 1 in 200 persons^1,2^. Accumulation of iron occurs in subjects with HH due to a homozygous C282Y substitution in the HFE gene product, which causes dysregulated hepatic hepcidin expression, resulting in unregulated iron absorption, leading to iron deposition in various tissues in the liver, heart and multiple other organs^3^. In a proportion of patients, untreated disease leads to end-organ damage, which includes the development of hepatic fibrosis or cirrhosis^4^. However, the phenotypic expression of iron overload is variable, with only a minority of patients with C282Y homozygosity developing symptoms and/or end organ damage related to severe iron overload^5^. In the liver, the diagnosis and identification of individuals with underlying advanced fibrosis or cirrhosis is crucial for prognosis, since these subjects exhibit poorer long-term survival related to a variety of complications of chronic liver disease, as well as markedly increased risk of hepatocellular carcinoma^6^. Known risk factors associated with an increased risk of advanced fibrosis or cirrhosis in HH include excessive alcohol consumption, serum ferritin levels greater than 1000 µg/L, male gender, the presence of diabetes mellitus and hepatic iron concentration (HIC) greater than 236 µmol/g^7–10^.

Interestingly, an earlier study suggested that advanced fibrosis could develop in individuals with lower HIC levels than usually considered to pose a risk, and that this may be related to longer duration of exposure to elevated hepatic iron^11^. This was confirmed by a subsequent study which demonstrated that the product of HIC and age improved the sensitivity and specificity for prediction of advanced fibrosis, when compared with HIC alone^12^.

While the measurement of HIC provides a quantitation of liver iron content, an approximation of total body mobilisable iron stores can be determined via therapeutic phlebotomy^13^. Each unit of blood (500 ml) removed during phlebotomy contains an estimated 200–250 mg of iron^14^. Thus, the total number of venesections required to achieve a serum ferritin level in the order 50–100 µg/L allows for estimation of total body iron stores. The aim of the current study was to examine the relationships between HIC, mobilisable iron (with and without duration of iron loading) and the prediction of advanced fibrosis, to ultimately determine whether mobilisable iron stores could provide a useful clinical predictor of advanced fibrosis in an independent large cohort of well-characterized subjects with HH and thereby assist in determining whether more specific tests for advanced fibrosis are required, such as liver biopsy.

## Patients and Methods

### Patients

We studied 202 consecutive subjects diagnosed with HH, and who underwent a liver biopsy at the Royal Brisbane and Women’s Hospital in Australia between 1972 and 2010 as part of routine standard of care for assessment of HH. Baseline demographic data, total number of venesections, alcohol consumption, biochemical results and liver biopsy histological assessments were available on all subjects. All subjects were confirmed as being C282Y homozygous on genetic testing. All subjects were routinely offered a liver biopsy as part of baseline assessment, and only those who declined did not receive one. Phlebotomy was performed weekly on all subjects until a serum ferritin level of less than 100 µg/L was achieved. Following this, patients had venesection at a frequency determined by the treating physician. Mobilisable iron was calculated using the total number of venesections required (one unit = 500 ml) to achieve a serum ferritin less than 100 µg/L multiplied by 250 mg of iron. Exclusion criteria included, age less than 16 years, or other forms of chronic liver disease such as chronic viral hepatitis, immune-mediated liver and metabolic liver diseases. The age of subjects was defined as the age at which the liver biopsy was performed. All subjects were untreated at the time of their study inclusion and had an alcohol consumption of <210 g per week for males, and 140 g per week for females. HIC was measured by atomic absorption spectrophotometry on fresh liver biopsy specimens^15^. Paraffin-embedded sections were stained with haematoxylin and eosin and Perls’ Prussian blue method and reviewed by liver histopathologists with expertise in HH who classified fibrosis stage according to the grading system of Scheuer: F0–no fibrosis, F1–mild fibrosis with enlarged portal tracts, F2–moderate periportal and portal-portal septa but intact architecture, F3–severe fibrosis with architectural distortion; and F4–cirrhosis with architectural distortion^16^. For the purposes of this study, subjects with hepatic fibrosis stages F3 and F4 were combined and termed ‘advanced fibrosis’. These studies were approved by the Human Research Ethics Committees of the Royal Brisbane and Women’s Hospital and the QIMR Berghofer Medical Research Institute, Brisbane, Australia and informed written consent was obtained at the time of entry into the study.

### Statistical analysis

All data are presented as the mean ± SEM unless otherwise specified. Student’s T-test or analysis of variance was used to analyse differences between groups and area under the receiver operator characteristic (AUROC) curve analysis was performed for evaluation of sensitivity and specificity of HIC (µmol/g), HIC × [age] (µmol/g.yr), mobilisable iron (g), mobilisable iron × [age] (g.yr) and serum ferritin (µg/L) parameters for the detection of advanced fibrosis. Fisher’s exact test was used to compare proportions of patients according to the severity of liver fibrosis and the presence or absence of advanced fibrosis. All statistical tests were conducted using GraphPad Prism 7 (GraphPad Software, San Diego, CA). All p-values are reported as two-sided and statistical significance was assigned for p < 0.05. Best sensitivity and specificity of the various tests was determined at the point where the two values were most similar. This would provide for the best rule in and rule out values for each test. Comparisons between ROC curves were performed using the method described by Hanley and McNeil^17^. Multivariable logistic regression analysis, including variables that were statistically significantly different in univariate analysis, was used to calculate the odds ratio (OR) for predictors of advanced fibrosis in subjects with HH.

## Results

The baseline study characteristics of all subjects are presented in Table 1. The mean subject age was 42.8 ± 1.1 years for males and 45.3 ± 1.8 years for females (p = 0.09). Sixty-eight percent of the study population were comprised of male subjects. The mean alcohol intake was significantly higher in males than females (p < 0.001), but within recommended safe consumption levels. Mean levels of HIC in the male group were significantly higher than that of the female group (209 ± 11.9 µmol/g versus 155.3 ± 14.3 µmol/g, p = 0.007), as were serum ferritin and transferrin saturation levels (Table 1). Advanced fibrosis (F3-F4) was more prevalent in male subjects. Age and alcohol intake were significantly higher in the subjects with advanced fibrosis (Table 2a). The mean values of HIC, HIC × [age], mobilisable iron, mobilisable iron × [age] and serum ferritin in the whole cohort (as well as male and female subgroups) were higher in the groups with advanced fibrosis (Table 2b).

**Table 1:** Baseline patient characteristics of HH subjects.

	Male	Female	p-value
N (%)	137 (67.8%)	65 (32.2%)	
Mean age	42.8 ± 1.13	45.3 ± 1.8	0.09
Mean HIC (µmol/g) Alcohol Intake (g/week) Serum Ferritin (µg/L) Transferrin Saturation (%)	209 ± 11.9 31.3 ± 3.3 1717 ± 109.4 83 ± 1.6	155.3 ± 14.3 16.9 ± 3.2 664 ± 74.9 76.9 ± 2.3	0.007 <0.001 <0.001 0.01
Fibrosis staging (%)			0.01
F0 F1 F2 F3 F4	65 (47.4) 19 (13.9) 16 (11.7) 10 (7.3) 27 (19.7)	47 (72.3) 8 (12.3) 5 (7.7) 1 (1.5) 4 (6.2)	

Data presented as mean ± standard error of mean or proportions. HIC, hepatic iron concentration.

**Table 2:** Mean age, alcohol intake, transferrin saturation and sex in HH subjects.

	All subjects (F0-2)	All subjects (F3-4)	p-value			
Age	42.2 ± 1.1	47.2 ± 1.6	0.04			
Alcohol intake (g/week)	21.1 ± 2.3	48.7 ± 7.5	<0.001			
Transferrin saturation (%)	80.7 ± 1.6	82.8 ± 2.4	0.53			
**Sex**						
Males	74%	26%	0.002			
Females	92%	8%				
**Mean HIC, HIC x [age], mobilisable iron, mobilisable iron x [age] and serum ferritin in HH subjects.**						
	**All subjects (F0-2)**	**All subjects (F3-4)**	**Males (F0-2)**	**Males (F3-4)**	**Females (F0-2)**	**Females (F3-4)**
Mean HIC (µmol/g)	139.5 ± 7.7	324 ± 22.7	150 ± 10.4	324 ± 23.7	113.5 ± 10.7	335 ± 83.4
p-value	<0.0001		<0.0001		0.0001	
HIC × [age] (µmol/g.yr)	5637 ± 384.2	13908 ± 1295	6019 ± 531.9	13598 ± 1333	5318 ± 501.4	22875 ± 3617
p-value	<0.0001		0.0002		<0.0001	
Mobilisable iron (g)	5 ± 0.3	15 ± 1.7	6.5 ± 0.4	15 ± 1.6	3.75 ± 0.3	18.75 ± 12.1
p-value	<0.0001		<0.0001		<0.0001	
Mobilisable iron × [age]	229.3 ± 14.3	778.2 ± 76.2	265.2 ± 20.6	777.6 ± 78	158 ± 13.4	778.2 ± 383.4
p-value	<0.0001		0.0001		<0.0001	
Serum ferritin (µg/L)	1181 ± 73.8	3142 ± 202.5	576.5 ± 56.1	2413 ± 431.3	951.2 ± 55.6	3088 ± 192.5
p-value	<0.0001		<0.0001		<0.0001	

Advanced fibrosis = F3-F4 fibrosis, No advanced fibrosis = F0-F2 fibrosis. Data presented as mean ± standard error of mean or proportions. HIC, hepatic iron concentration.

The ROC curve analysis for the diagnosis of advanced fibrosis in HH males and females combined demonstrated that HIC alone had equivalent optimum sensitivity and specificity values of 73%, with a cut-off HIC level of greater than 200 µmol/g (AUROC 0.83) (Table 3). In the analysis of male subjects alone, the AUROC was 0.79, with an optimum sensitivity and specificity of 74% and 68%, respectively, with a cut-off HIC level of greater than 200µmol/g. In the analysis of female subjects, the AUROC was 0.93, with an equivalent optimum sensitivity and specificity both of 80% with a cut-off HIC of greater than 197 µmol/g.

**Table 3:** ROC analysis reporting AUROC, sensitivity, specificity for HIC (µmol/g); HIC × [age] (µmol/g.yr); Mobilisable iron (g); Mobilisable iron × [age] (g.yr); serum ferritin (µg/L) in the detection of advanced hepatic fibrosis in HH subjects.

	AUROC	Sensitivity	Specificity	Cut-off	z	p-value
**HIC**						
All subjects Male Female	0.83 (0.73–0.89) 0.79 (0.69–0.86) 0.93 (0.81–1.00)	73% 74% 80%	73% 68% 80%	>200 >200 >197	1.956	0.050
**HIC** × **[age]**						
All subjects Male Female	0.82 (0.76–0.90) 0.79 (0.74–0.90) 0.97 (0.67–1.00)	73% 70% 100%	74% 70% 87%	>9183 >9152 >10400	1.982	0.047
**Mobilisable iron**						
All subjects Male Female	0.92 (0.88–0.96) 0.88 (0.82–0.94) 0.99 (0.98–1.00)	83% 79% 79%	83% 79% 79%	9.6 10.8 9.9		
**Mobilisable iron** × **[age]**						
All subjects Male Female	0.93 (0.88–0.97) 0.89 (0.83–0.95) 1.00 (1.00–1.00)	87% 79% 100%	88% 79% 100%	467 495 577	0.759	0.448
**Serum Ferritin**						
All subjects Male Female	0.94 (0.91–0.97) 0.92 (0.87–0.96) 0.99 (0.96–1.00)	85% 82% 100%	85% 81% 98%	>1665 >1826 >1665	0.472	0.637

95% confidence intervals are show in brackets. The AUROC for mobilisable iron was compared with each of the other parameters using the method of Hanley and McNeil, generating a z-score and p-value^17^. AUROC, area under the receiver operator characteristic; HIC, hepatic iron concentration.

The product of HIC and age allowed for the incorporation of the duration of exposure to iron into the prediction of advanced fibrosis. The ROC curve analysis demonstrated that HIC × [age] had an optimum sensitivity and specificity of 73% and 74%, respectively, for the diagnosis of advanced liver fibrosis in HH in males and females combined (Table 3), with a cut-off HIC × [age] of greater than 9183 µmol/g.yr (AUROC 0.82). The optimum sensitivity and specificity in male subjects were both 70% (AUROC 0.79), with a cut-off HIC × [age] of greater than 9152 µmol/g.yr. The optimum sensitivity and specificity in female subjects was 100% and 87% (AUROC 0.97), respectively, with a cut-off HIC × [age] of greater than 10400 µmol/g.yr.

The ROC curve analysis for mobilisable iron in all subjects demonstrated an AUROC of 0.92, with sensitivity and specificity both of 83%, with a cut-off of more than 9.6 g for the prediction of advanced fibrosis (Table 3). In male subjects, both the sensitivity and specificity were 79% with a cut-off of more than 10.8 g (AUROC 0.88). In female subjects, both the sensitivity and specificity were 79% with a cut-off of more than 9.9 g (AUROC 0.99).

The ROC curve analysis for the product of mobilisable iron × [age] in all subjects demonstrated a sensitivity and specificity of 87% and 88%, respectively, with a cut-off of 467 g.yr (AUROC 0.93) for the prediction of advanced fibrosis (Table 3). In male subjects both the sensitivity and specificity were 79% using a cut-off of more than 495 g.yr (AUROC 0.89). In female subjects, both the sensitivity and specificity were 100% using a cut-off of more than 577 g.yr (AUROC 1.00).

The ROC curve analysis for serum ferritin in all subjects demonstrated an equivalent sensitivity and specificity of 85%, with a cut-off of more than 1665 µg/L (AUROC 0.94) for the prediction of advanced fibrosis (Table 3). In male subjects the sensitivity and specificity were 82% and 81% respectively, using a cut-off of more than 1826 µg/L (AUROC 0.92). In female subjects, the sensitivity and specificity were 100% and 98% respectively, using a cut-off of more than 1665 µg/L (AUROC 0.99).

ROC curves for mobilisable iron, HIC, HIC × [age], mobilisable iron × [age] and serum ferritin were compared using the method of Hanley and McNeil^17^. Mobilisable iron was significantly better than either HIC or HIC × [age] (Table 3). There were no significant differences between mobilisable iron and either mobilisable iron × [age] or serum ferritin levels.

**Figure 1:**
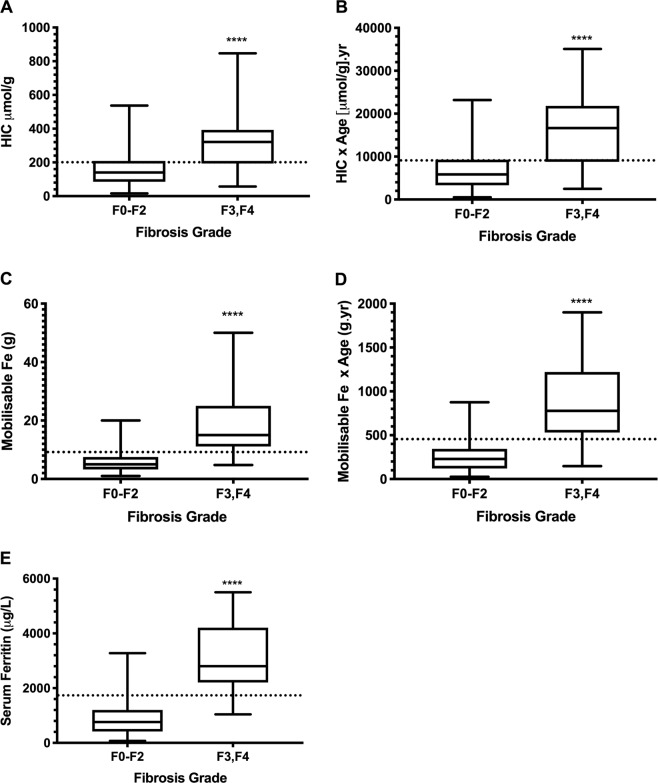
Box and whisker plot demonstrating mean values for all subjects, with or without advanced fibrosis with proposed cut-off levels for predicting advanced fibrosis (F3-F4) in HH patients (dotted lines). For HIC (**A**), HIC × age (**B**), mobilisable iron (**C**), mobilisable iron × age (**D**) and serum ferritin (**E**).

**Figure 2:**
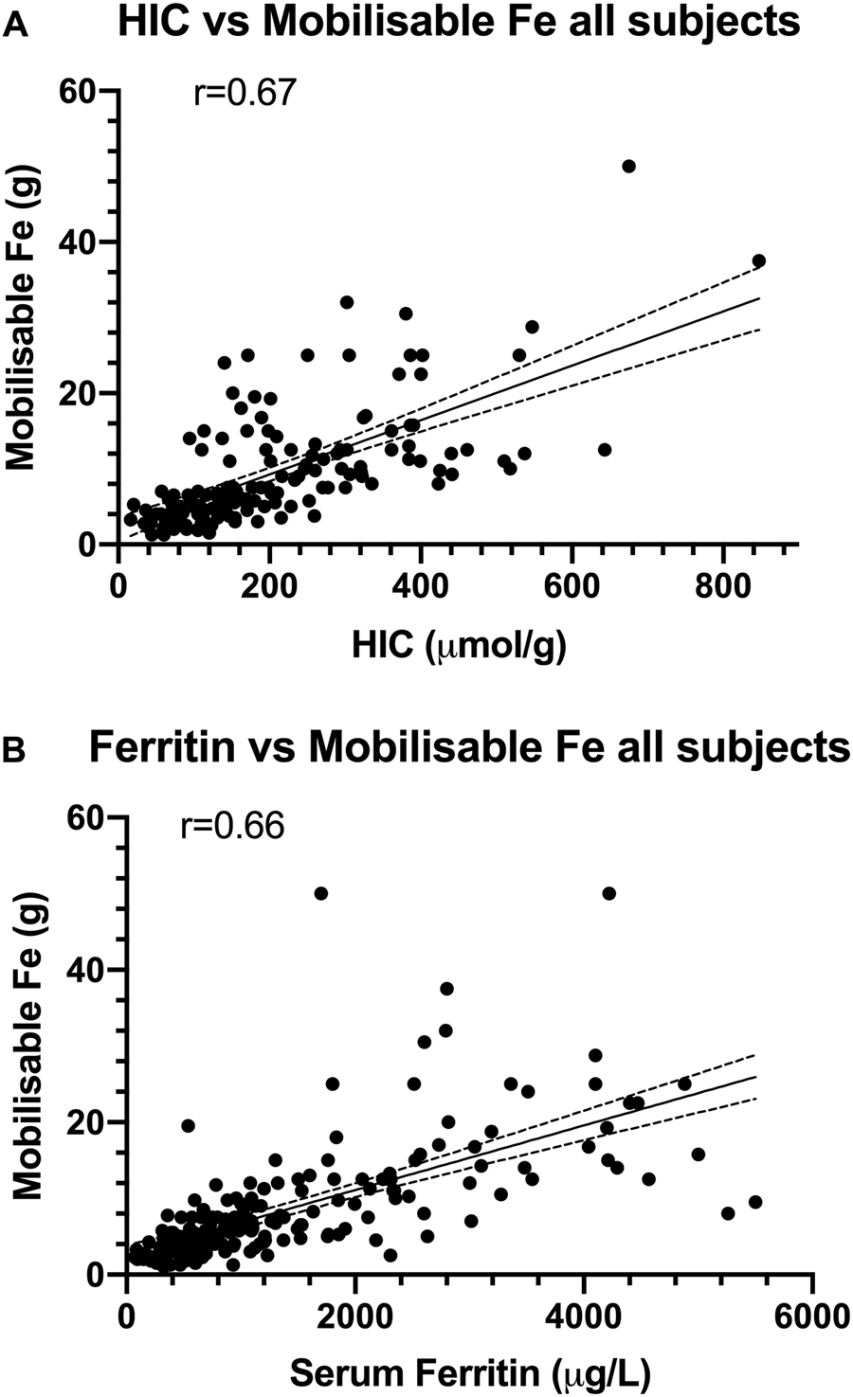
Correlation between (**A**) HIC and mobilisable and (**B**) serum ferritin level and mobilisable iron. The 95% confidence intervals are shown on the graphs as dotted lines.

The mean values of HIC, HIC × [age], mobilisable iron, mobilisable iron × [age] and serum ferritin are presented in box and whisker plots (Fig. 1), highlighting proposed cut-off levels for predicting advanced fibrosis. Figure 2 shows the similarly significant correlations between mobilisable iron and HIC (r = 0.67) and between mobilisable iron and serum ferritin (r = 0.66). A multivariable logistic regression analysis was undertaken for all subjects with HH examining age, HIC, alcohol intake, serum ferritin and mobilisable iron. Mobilisable iron was shown to be a significant predictor of advanced fibrosis, after adjusting for age, sex, alcohol intake, HIC and serum ferritin (Table 4).

**Table 4:** Multivariable logistic regression analysis of factors associated with prediction of advanced fibrosis in all subjects with HH.

	Odds Ratio (95% confidence intervals)	*p(unitalicize the p please)*
Alcohol intake	1.02 (1.004–1.029)	0.01
Age	1.042 (1.006–1.079)	0.02
HIC	1.009 (1.006–1.013)	<0.001
Mobilisable iron	1.064 (1.011–1.120)	0.02

Adjusted for serum ferritin and sex.

## Discussion

HH is a common disease that has become more widely recognized since genotyping for the HFE mutation commenced in 1996. A majority of patients are now diagnosed earlier, well in advance of the development of overt symptoms or systemic complications, and many of them are being managed in the primary care setting by general practitioners. However, some patients are unfortunately diagnosed late with quite advanced disease, largely due to a low index of suspicion on the part of some primary care physicians and the fact that the key screening test for iron overload (serum ferritin levels) is not included in the standard medical biochemical analysis. Undiagnosed, and thus untreated, patients are at risk of significant life limiting complications of cirrhosis, as well as HCC. Thus the ongoing clinical challenge in patients with HH has been to stratify risk for iron overload and advanced fibrosis. Staging of hepatic fibrosis is traditionally established through assessment of liver histology requiring patients to accept the potential risks and complications of a biopsy. Additionally, the measurement of HIC via magnetic resonance imaging (MRI) has been shown to correlate well with liver biopsy-derived HIC, making the assessment of liver iron levels much less invasive, and potentially more accessible to patients^12,18^. The use of non-invasive measurements of fibrosis in other chronic liver diseases through MRI, elastography, or serum based biomarkers, has increased globally with increased availability, and reduced costs. These modalities have primarily been validated in patients with chronic viral hepatitis and non-alcoholic fatty liver disease. While some groups have attempted to assess the utility of a variety of different non-invasive biomarkers in patients with HH, most studies lack rigor due to inadequate sample size or the absence of biopsy validated fibrosis and the cut-off values for prediction of advanced fibrosis have not been clearly defined^19^. Unfortunately, potentially efficacious modalities such as MRI elastography are not easily accessible outside of tertiary health care facilities and have associated high costs and limited availability. A study by Beaton *et al*. had demonstrated that prediction of cirrhosis could be performed reliably in the majority of patients utilizing the clinical parameters of serum ferritin above 1000 µg/L, with an elevated aminotransferase level and a platelet count of less than 200 × 10^9^/L^8^. However, recent studies have suggested that approximately 30–64% of patients with cirrhosis do not fulfil all three criteria^8,20^. This could result in potential missed diagnosis and would still require patients with serum ferritin greater than 1000 µg/L to undergo liver biopsy.

In this current study, we investigated the relationship between HIC, age, serum ferritin and mobilisable iron in the prediction of advanced fibrosis to confirm, and extend an earlier study which demonstrated the product of HIC and age improved accuracy for the prediction of advanced fibrosis in patients with HH^12^. Our results suggest that the effect of duration of exposure to elevated hepatic iron stores was more important in women for the prediction of advanced fibrosis (F3-F4). A sub-analysis of our data for the prediction of F2-F4 fibrosis demonstrated that the accuracy of HIC is similarly improved by the addition of age, albeit to a lesser degree (Supplementary Table S1). The more significant influence of duration of exposure in women could be related to gender-related differences in rates of iron accumulation during adult life, that is, women have lower HIC levels prior to menopause with an exponential increase following menopause in women^21^.

Furthermore, we have demonstrated that mobilisable iron is an accurate method for retrospectively determining advanced fibrosis, independent of serum ferritin and HIC. It is also less susceptible to the effects of age (presumably because mobilisable iron in itself is a fairly accurate test). Additionally, comparison of the ROC curves demonstrated that mobilisable iron was at the least an equivalent test compared with mobilisable iron × [age] or serum ferritin levels. Mobilisable iron was superior to both HIC and HIC × [age] in predicting advanced hepatic fibrosis. A prior study by Phatak *et al*. also showed that mobilisable iron was an accurate and reliable surrogate for total body iron^13^. One potential limitation of using mobilisable iron to predict risk of underlying advanced fibrosis is in the setting of significant or chronic blood loss. We found that there was significant correlation between HIC and mobilisable iron with an R^2^ of 0.45 (r = 0.67, p < 0.0001) which is consistent with previous studies^13,22^. To assess the effect of co-factors, we performed a multivariable analysis which demonstrated that the association of mobilisable iron and age with advanced fibrosis was independent of alcohol intake. Furthermore, mean alcohol intake in the study population was well below the threshold generally considered significant for alcohol-related liver disease as per our inclusion criteria.

Limitations of our study include the retrospective design and absence of data regarding body mass index (BMI) in a number of subjects. Additionally, while a majority of competing risk factors for hepatic fibrosis were excluded in our subjects, complete data regarding steatosis or steatohepatitis assessment on histology was not available as a number of cases were historical, before formalization of scoring systems were routinely implemented. Additionally, although this cohort contained a relatively low number of subjects with advanced fibrosis, this study represents one of the largest cohorts of subjects with HH and biopsy-staged hepatic fibrosis providing confidence in the robustness of the data reported.

## Conclusion

Measurement of body iron stores by mobilisable iron (±age) can be used to predict the likelihood of significant hepatic fibrosis in HH and thus justify the need for more specific, but costly and invasive tests such as liver biopsy. Mobilisable iron stores of greater than 9.9 g in women and 10.8 g in men demonstrates high utility in the prediction of advanced fibrosis. This can be used to guide appropriate stratification of management for potential complications of chronic liver disease.

## Supplementary information

Supporting Information.

## Data Availability

The datasets generated during and/or analysed during the current study are available from the corresponding author on reasonable request.
