# Supplementary material for: Utility of hepatic or total body iron burden in the assessment of advanced hepatic fibrosis in HFE hemochromatosis
Source: Sci Rep. 2019 Dec 27;9:20234. doi: 10.1038/s41598-019-56732-0 (PMC6934689; doi:10.1038/s41598-019-56732-0)
Supplement: Supplementary file 1 — Supporting Information. [file 41598_2019_56732_MOESM1_ESM.docx]

**SUPPLEMENTARY TABLE**

**Table S1.** ROC analysis reporting AUROC, sensitivity, specificity for HIC (µmol/g); HIC x [age] (µmol/g.yr); Mobilisable iron (g); Mobilisable iron x [age] (g.yr) in the detection of advanced hepatic fibrosis in HH subjects using an alternate cut off defining advanced fibrosis as F2-F4 fibrosis.

|  | AUROC | Sensitivity | Specificity | Cutoff | p-value |
| --- | --- | --- | --- | --- | --- |
| HIC  All subjects  Male  Female | 0.72  0.68  0.79 | 67%  68%  67% | 67%  68%  67% | >177.5  >188.5  >147.5 | < 0.0001  0.0004  0.004 |
| HIC x [age]  All subjects  Male  Female | 0.74  0.72  0.86 | 65%  66%  67% | 65%  66%  67% | >8018  >7834  >8166 | 0.0001  < 0.0001  0.0004 |
| Mobilisable iron  All subjects  Male  Female | 0.84  0.81  0.77 | 76%  75%  67% | 74%  74%  67% | >7.4  >8.75  >4.6 | < 0.0001  < 0.0001  0.03 |
| Mobilisable iron x [age]  All subjects  Male  Female | 0.83  0.80  0.84 | 76%  73%  83% | 75%  73%  83% | >327  >365  >278 | < 0.0001  < 0.0001  0.007 |

AUROC, area under the receiver operator characteristic; HIC, hepatic iron concentration.
